# It is not just about transcription: involvement of brain RNA splicing in substance use disorders

**DOI:** 10.1007/s00702-024-02740-y

**Published:** 2024-02-24

**Authors:** Luana Carvalho, Amy W. Lasek

**Affiliations:** Department of Pharmacology and Toxicology, Virginia Commonwealth University, 1220 E. Broad ST, Box 980613, Richmond, VA 23298 USA

**Keywords:** Alternative splicing, Addiction, Substance use disorder, Alcohol, Cocaine, Splicing factor, Spliceosome

## Abstract

Alternative splicing is a co-transcriptional process that significantly contributes to the molecular landscape of the cell. It plays a multifaceted role in shaping gene transcription, protein diversity, and functional adaptability in response to environmental cues. Recent studies demonstrate that drugs of abuse have a profound impact on alternative splicing patterns within different brain regions. Drugs like alcohol and cocaine modify the expression of genes responsible for encoding splicing factors, thereby influencing alternative splicing of crucial genes involved in neurotransmission, neurogenesis, and neuroinflammation. Notable examples of these alterations include alcohol-induced changes in splicing factors such as HSPA6 and PCBP1, as well as cocaine's impact on PTBP1 and SRSF11. Beyond the immediate effects of drug exposure, recent research has shed light on the role of alternative splicing in contributing to the risk of substance use disorders (SUDs). This is exemplified by exon skipping events in key genes like *ELOVL7*, which can elevate the risk of alcohol use disorder. Lastly, drugs of abuse can induce splicing alterations through epigenetic modifications. For example, cocaine exposure leads to alterations in levels of trimethylated lysine 36 of histone H3, which exhibits a robust association with alternative splicing and serves as a reliable predictor for exon exclusion. In summary, alternative splicing has emerged as a critical player in the complex interplay between drugs of abuse and the brain, offering insights into the molecular underpinnings of SUDs.

## Introduction

Alternative splicing is a crucial co-transcriptional process that allows for a single gene to produce multiple protein isoforms with distinct functional properties. This phenomenon plays a significant role in regulating transcription and increasing protein diversity in various tissues and cell types. Evidence from many species indicates that substance use can lead to alternatively spliced transcripts in different brain regions, contributing to the complex molecular changes associated with the transition to substance misuse. This review explores the relationship between alternative splicing and SUDs, shedding light on the molecular mechanisms of drug-induced changes in the brain that contribute to these disorders. The exploration of drug-induced changes in alternative splicing represents a recent and evolving field of study. The primary goal of this review is to provide a summary of the literature in this burgeoning research area and highlight new technologies that have expanded our knowledge of mechanisms involved in alternative splicing.

## RNA splicing

Most genes in higher eukaryotes are transcribed as pre-messenger RNA (pre-mRNA), which undergoes splicing as it is transcribed from the DNA template. The process of RNA splicing involves the removal of introns and the ligation of exons to form mature mRNA (Wilkinson et al. 2020). RNA splicing is enabled by the spliceosome, a megadalton machine composed of uridine-rich small nuclear RNA (snRNA: U1, U2, U4, U5, and U6), proteins from the NineTeen complex (NTC), the NTC-related complex (NTR), RNA binding proteins (RBPs) and RNA-dependent ATPase/helicases (Wilkinson et al. 2020; Ule and Blencowe 2019). RBPs in the spliceosome are commonly referred to as splicing factors and play a pivotal role in determining the specificity of splice site selection and the formation of the exon definition complex (the complex of RNA binding proteins and regulatory elements that define exon boundaries) by recognizing specific sequences within the precursor mRNA. These sequences are known as splicing enhancers or silencers. RBPs bound to splicing enhancers stabilize the assembly of the spliceosome and promote exon splicing (Wan et al. 2019; Matera and Wang 2014). Conversely, when RBPs bind to splicing silencers, they impede the assembly of the spliceosome, leading to the inhibition of exon definition complex formation.

Alternative splicing involves the use of alternative splice sites to join exons in various combinations, thereby generating multiple transcripts from a single gene. Alternatively, spliced transcripts can generate diverse protein isoforms with distinct cellular functions through the inclusion of alternative functional domains (Sibley et al. 2016). Alternative splicing results in events such as exon skipping, in which an exon is excluded in the mature mRNA (Wan et al. 2019), the use of mutually exclusive exons, in which only one exon from a cluster is included, intron retention, in which the intron is not spliced out (Galante et al. 2004), and the use of alternative 3′ and 5′ splice sites located within an intron or exon (Blencowe 2006). Alternative splicing is a fundamental cellular process with dual implications for health, serving as a crucial mechanism for normal physiological functions but also playing a role in the pathogenesis of disease. Alternative splicing enhances genomic and protein diversity, which is essential for tissue-specific functions and overall cellular homeostasis. For instance, in the nervous system, alternative splicing ensures proper neuronal development, neuronal migration, axon guidance, and synaptogenesis (Zhou et al. 2018; Raj and Blencowe 2015). However, when alternative splicing becomes dysregulated, it may lead to the production of dysfunctional proteins or disrupt vital cellular processes, contributing to the onset and progression of neurological and psychiatric diseases (Nik and Bowman 2019). Studies indicate that exposure to drugs of abuse results in alternatively spliced transcripts in different brain regions, potentially contributing to the development of SUDs (Van Booven et al. 2021; Xu et al. 2014, 2021; Piltonen et al. 2021; Huggett et al. 2022; Li et al. 2023; Krapacher et al. 2022).

## Drug-induced changes in RNA splicing

**Fig. 1:**
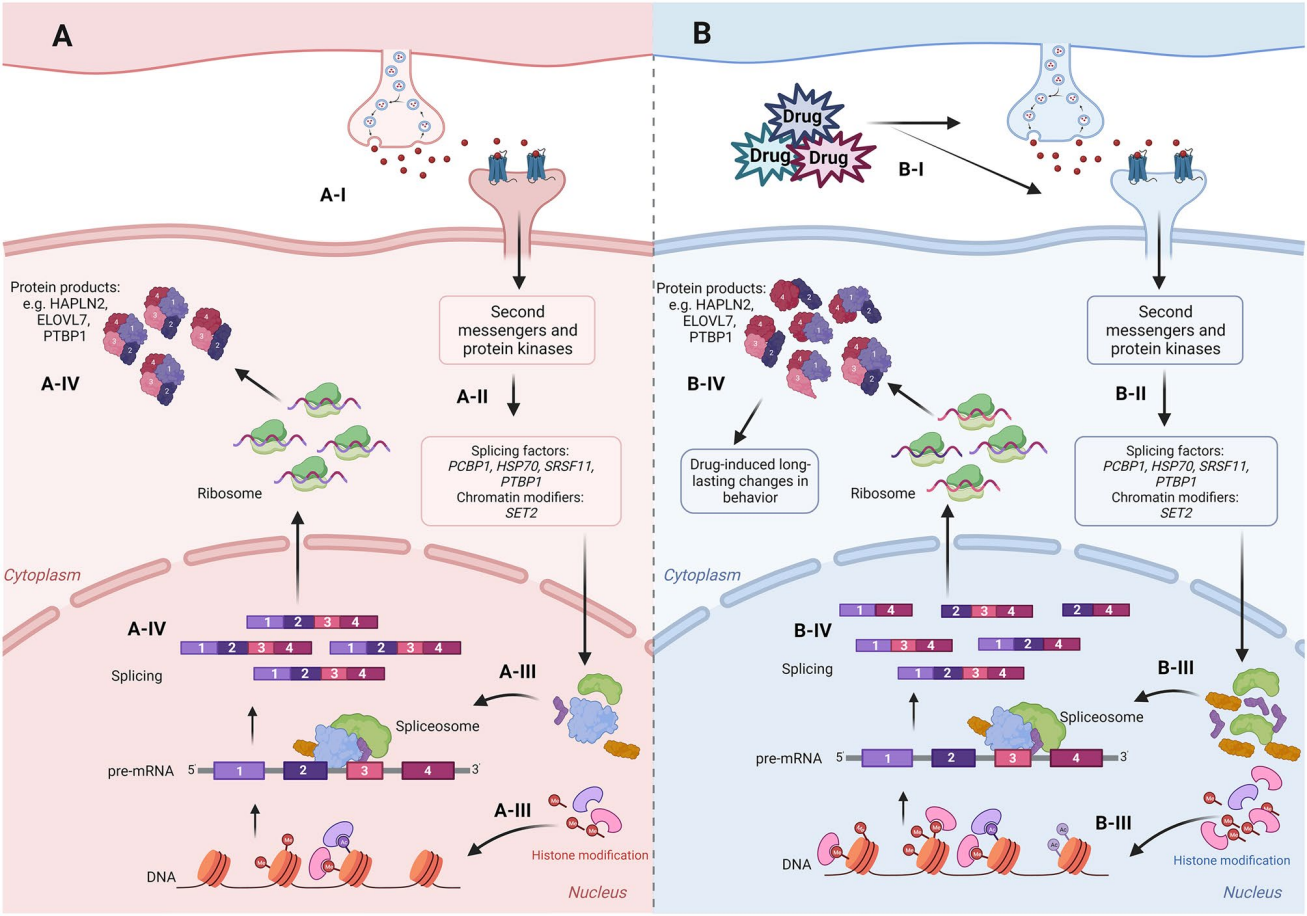
Drug-induced changes in RNA splicing. Illustrations depict neuronal signaling in the **A** absence and **B** presence of drugs of abuse. Generally, drugs of abuse can regulate intracellular neuronal signaling cascades by targeting synaptic components, including neurotransmitter receptors and reuptake mechanisms (B-I). The activation or inhibition of these specific signaling pathways, through second messengers and protein kinases, leads to the upregulation or downregulation of genes encoding splicing factors (such as PCBP1, HSP70, SRSF11, and PTBP1) and chromatin modifiers (such as SET2) (B-II). Altered levels of splicing factors and histone modifications can further impair splicing, for example, by influencing splice site selection or altering splice site accessibility (B-III). Ultimately, alternatively spliced transcripts are translated into proteins, which may or may not be functional, further contributing to drug-induced long-lasting changes in behavior (B-IV). In contrast, in the absence of drugs of abuse, optimal levels of splicing factors and chromatin modifications (A-III) lead to canonical splicing and the translation of functional proteins (A-IV). Figure created with Biorender.com

In recent years, researchers have been exploring how repeated drug exposure can influence splicing patterns and how these alterations might contribute to the physiological and behavioral effects associated with drug use. Table 1 lists studies on drug-induced changes in RNA splicing. One way in which drugs of abuse can impact RNA splicing is by altering the expression of genes encoding splicing factors (Van Booven et al. 2021; Carvalho et al. 2023). Psychotropic drugs exert their effects by targeting synaptic elements, ion channels, and neurotransmitter receptors, subsequently modifying intracellular signaling cascades (Nestler and Lüscher 2019; Robison and Nestler 2011). The activation or inhibition of these specific signaling pathways, in turn, leads to the upregulation or downregulation of genes encoding splicing factors. Altered protein levels of splicing factors can then exert regulatory control over alternative splicing in genes responsible for encoding neurotransmitter receptors, transporters, and other essential proteins involved in synaptic transmission or glial cell function, ultimately contributing to drug-related changes in neuronal signaling and function, and behavior (Fig. 1).

**Table 1:** Published studies reporting drug-induced changes in RNA splicing

Drug	Organism	Brain region	References
Alcohol	Human	Fetal cortical slices	Kawasawa et al. (2017)
Alcohol	*Drosophila melanogaster*	Mushroom body	Petruccelli et al. (2018)
Alcohol	*Drosophila melanogaster*	Whole body	Signor and Nuzhdin (2018)
Alcohol	DBA/2J mice	Prefrontal cortex	O'Brien et al. (2018)
Alcohol	C57BL/6NCrl mice	Hippocampus	Wolfe et al. (2019)
Alcohol	Cell culture	Human fetal neuronal culture	Donadoni et al. (2019)
Alcohol	Rhesus macaques C57BL/6J mice	Medial prefrontal cortex	Bogenpohl et al. (2019)
Alcohol	*Drosophila melanogaster*	Mushroom body	Petruccelli et al. (2020)
Alcohol	Human	Superior frontal cortex Nucleus accumbens Basolateral amygdala Central nucleus of the amygdala	Van Booven et al. (2021)
Alcohol	*Lrap* transgenic Wistar rat	Whole brain	Saba et al. (2021)
Alcohol	HXB/BXH Recombinant Inbred Rat Panel	Whole brain	Lusk et al. (2022)
Alcohol	Rhesus macaques Human	Human: Superior frontal cortex Nucleus accumbens Basolateral amygdala Central nucleus of the amygdala Rhesus macaques: Nucleus accumbens Central nucleus of the amygdala	Huggett et al. (2023)
Alcohol	Human	Dorsolateral prefrontal cortex	Li et al. (2023)
Alcohol	Sprague Dawley rats	Dorsal hippocampus	Carvalho et al. (2023)
Cocaine	C57BL/6J mice	Nucleus accumbens	Feng et al. (2014)
Cocaine	C57BL/6J mice	Nucleus accumbens	Cates et al. (2018)
Cocaine	Gad67^Cre^ mice Nkx2.1^Cre^ mice Gpr101^Cre^ mice Alk4^fl/fl^ mice	Nucleus accumbens	Krapacher et al. (2022)
Cocaine	C57BL/6J mice	Nucleus accumbens	Xu et al. (2021)
Morphine	C57BL/6J mice	Trigeminal ganglia Nucleus accumbens	Zhang et al. (2021)
Fentanyl Heroin Oxycodone	Human	Dorsal-lateral prefrontal cortex Nucleus accumbens Ventral midbrain	Huggett et al. (2022)

### Alcohol

A growing number of studies have reported that alcohol exposure results in differential alternative splicing across different species (Lusk et al. 2022; Saba et al. 2021). For example, Signor and Nuzdin (Signor and Nuzhdin 2018) demonstrated, using RNA sequencing (RNA-Seq) in the fruit fly *Drosophila melanogaster*, that alterations in alternative splicing actively occur and are subject to regulation or modulation in response to acute alcohol exposure. Another RNA-Seq study in fruit flies found that associative training of alcohol exposure with an odor cue switched usage of specific isoforms of dopamine 2 receptor (*Dop2R*) within *Drosophila* memory-encoding mushroom body neurons (Petruccelli et al. 2018). RNA-Seq from human fetal cortical tissue exposed to ethanol showed significant changes in alternative splicing of genes associated with cell death and apoptosis (Kawasawa et al. 2017). In mice, acute ethanol treatment altered the exon usage of genes that significantly overlapped with changes observed in mice treated with an NMDAR antagonist, Ro 25-6981. As alcohol and Ro 25-6981 can both have rapid antidepressant effects, these results implicate alternative splicing as a potential mechanism for the behavioral effects (Wolfe et al. 2019).

Recently, a genome-wide RNA sequencing (RNA-Seq) study was conducted in different human postmortem brain regions from individuals diagnosed with alcohol use disorder (AUD). The superior frontal cortex, nucleus accumbens (NAc), basolateral amygdala and central nucleus of the amygdala were examined for differential RNA splicing between control and AUD subjects using bioinformatics tools (Van Booven et al. 2021). The study revealed a higher number of differential splicing events compared to changes in mRNA levels. Specifically, most of these splicing events resulted from exon skipping and alterations in the levels of mutually exclusive exons. To elucidate the underlying mechanism behind these differential splicing events, the authors first examined whether snRNA transcripts (U1, U4, U6, and U7) were changed in the brains of AUD subjects compared with controls, but they did not find differences in levels of snRNAs. Next, they measured levels of the mRNAs encoding protein components of the spliceosome. Notably, they observed a substantial increase in the transcript for splicing factor heat shock protein family A (Hsp70) member 6 (*HSPA6*) (Van Booven et al. 2021). The differentially spliced RNA targets of HSPA6 in AUD have yet to be identified, but this is an important area for future investigation.

In rat hippocampus, withdrawal from chronic alcohol exposure in males increased levels of mRNAs for genes encoding components of the RNA splicing machinery and changed RNA splicing (Carvalho et al. 2023). Specifically, mRNA levels of the splicing factor poly r(C) binding protein 1 (PCBP1) increased in the hippocampus of alcohol-withdrawn rats compared with controls. Remarkably, *PCBP1* mRNA was also increased in the postmortem hippocampus of human subjects diagnosed with AUD compared to control subjects (Carvalho et al. 2023). RNA immunoprecipitation demonstrated enriched PCBP1 binding around an alternative splice site in exon 4 of the *Hapln2* pre-mRNA in both ethanol-withdrawn rats and individuals diagnosed with AUD (Carvalho et al. 2023). Ethanol withdrawal also resulted in increased usage of the alternative splice site in *Hapln2* exon 4. The use of this splice site is predicted to introduce a frameshift and stop codon, which could result in the expression of a truncated protein or nonsense-mediated decay of the transcript. HAPLN2 is an extracellular matrix protein located at the nodes of Ranvier in myelinated white matter. Its role is to maintain the extracellular diffusion barrier, ensuring proper nerve conduction velocity (Bekku et al. 2010; Rasband and Peles 2021). Differential alternative splicing of *Hapln2* in response to chronic alcohol exposure could therefore negatively impact neurotransmission.

One concern with chronic alcohol exposure is neurotoxicity, leading to cognitive deficits and increased risk of developing neurodegenerative conditions such as Alzheimer's and Parkinson's diseases (Visontay et al. 2021). Interestingly, alcohol-mediated toxicity appears to be linked with different splice variants. In neuroblastoma cells, alcohol exposure reduced protein levels of serine/arginine-rich splicing factor 1 (SRSF1) and shifted the alternative splicing of the MCL1 antiapoptotic protein towards the shorter isoform (Mcl-1S) over the long isoform (Mcl-1L) (Sariyer et al. 2017). The longer isoform enhances cell survival by inhibiting apoptosis, while the shorter isoform may promote apoptosis in response to alcohol exposure. To confirm the role of SRSF1 downregulation in the suppression of the longer MCL-1L isoform, the authors showed that overexpression of SRSF1 recovered the expression of MCL-1L, suggesting that ethanol-mediated suppression of SRSF1 expression is indeed involved in MCL-1L downregulation and may contribute to ethanol-induced toxicity (Sariyer et al. 2017).

In a follow-up study, Donadoni et al. (2019) showed that neuronal progenitors and immature neurons cultured from fetal brain tissue are highly sensitive to the toxic effects of ethanol, wherein a significant decrease in the Mcl-1L/Mcl-1S ratio in a dose- and time-dependent manner was observed in comparison to mature neurons (Donadoni et al. 2019). Interestingly, ectopic expression of Mcl-1L isoform in neural progenitors was able to recover the viability loss and apoptosis induced by alcohol exposure (Donadoni et al. 2019). Together, these results raise the possibility that alternative splicing of Mcl-1 may play a role in the mechanism underlying ethanol-induced neurotoxicity (Donadoni et al. 2019; Sariyer et al. 2017).

Alterations in the expression of splicing factors may contribute not only to neurotransmission impairment and neurotoxicity during alcohol exposure but also to the development of ethanol-related behaviors. Repeated alcohol exposure can lead to the formation of long-lasting memories associated with sensory cues related to intoxication, which can trigger relapse in individuals attempting to quit drinking. In *Drosophila melanogaster*, differential transcript isoforms were found in the mushroom body of flies trained with odor cues associated with ethanol compared with ethanol exposure alone, and with odor cues alone. The importance of alternative splicing in ethanol-associated memory was demonstrated by the knockdown of spliceosome-associated proteins in mushroom body neurons, which prevented the formation of ethanol-associated memories (Petruccelli et al. 2020).

These findings raise questions about whether alcohol-induced changes in splicing factors might also influence other behaviors like craving and negative affective states related to alcohol consumption and withdrawal, such as anxiety and depression in humans. This prompts a broader discussion regarding whether our studies are overly focused on transcriptional changes at the expense of examining the functional role of RNA splicing on behavior.

Finally, recent studies have highlighted that alternative splicing may contribute to the risk of AUD, in addition to being altered by alcohol exposure (Li et al. 2023; Lusk et al. 2022; Huggett et al. 2022). In a recent study, Huggett et al. (2023) utilized RNA-Seq data from three brain regions (prefrontal cortex, nucleus accumbens, and amygdala) in individuals diagnosed with AUD (*n* = 56; ages 40–73; 100% ‘Caucasian’). They also employed genome-wide association data on AUD (*n* = 435,563, ages 22–90; 100% European American) to explore the genetic mechanisms of alternative splicing in AUD. The findings revealed more than 700 differentially spliced genes between individuals with AUD and controls, along with over 6000 splicing quantitative trait loci (sQTL) associated with 170 of the 700 genes (Huggett et al. 2023). Some of these genes are involved in drug metabolism (*CYP2C19* and *CYP2C9*), intracellular signaling (*GRK4*, *GRK6*, *HDAC3*, *PRKACB*, and *MAPK3K6*), and ion channels (*CACNA1A*, *CACNA1G*, *CACNB2*, and *KCNMA1*). The authors reported specific SNPs linked to altered splicing events and suggested that DNA variants in and around these differentially spliced genes could contribute to the heritability of AUD.

In a separate study, a Mendelian randomization-based approach applied to the Collaborative Studies on the Genetics of Alcoholism (COGA) data identified 27 exon-skipping events predicted to influence AUD risk. For instance, the skipping of the second exon within the ELOVL fatty acid elongase 7 (*ELOVL7*) gene showed strong associations with alcohol dependence and problematic drinking (Li et al. 2023). This exon was also found to contribute to alterations in gray matter volumes across various brain regions, including the visual cortex, a region implicated in AUD (Li et al. 2023). In rats, a predisposition to voluntary alcohol consumption trait in the two-bottle choice paradigm was associated with specific isoforms from genes related to brain inflammation and the immune response (e.g., *Lrap*, *Ift81*, and *P2rx4*) (Lusk et al. 2022). Collectively, these findings indicate a genetic contribution of alternative splicing to AUD.

### Cocaine

Studies have demonstrated that repeated exposure to cocaine can lead to changes in RNA splicing and to changes in isoform abundance within the nucleus accumbens (Feng et al. 2014; Cates et al. 2018; Krapacher et al. 2022; Xu et al. 2021). Through transcriptomics, Cates et al. (2018) demonstrated increased mRNA levels of *E2f3a*, an isoform of the *E2f3* transcription factor*,* in the mouse nucleus accumbens after repeated cocaine injections. Interestingly, when *E2F3a* was overexpressed in the nucleus accumbens, it resulted in similar patterns of both mRNA transcript levels and alternative splicing events as seen after chronic cocaine treatment. One of the genes that underwent alternative splicing in this context was *Ptbp1*, which encodes the splicing factor polypyrimidine tract binding protein 1. Overexpression of E2F3a increased the inclusion rate of *Ptbp1* exon 8, leading to the insertion of 26 amino acids into its RNA binding domain. Notably, E2F3a DNA binding was enriched at the *Ptbp1* splice site, suggesting that E2F3a can regulate alternative splicing, but the exact mechanism is currently unknown. These results indicate that PTBP1 may mediate the effects of E2F3a on cocaine-induced alternative splicing in the nucleus accumbens.

In addition to its role in alternative splicing during withdrawal from chronic alcohol exposure (Carvalho et al. 2023), PCBP1 also appears to play a role in the alternative splicing of the FosB gene in response to cocaine (Krapacher et al. 2022). Following repeated dopamine receptor stimulation mimicking cocaine sensitization in vitro, the activation of the dopamine D1 receptor signaling pathway synergized with the activin/ALK4/Smad3 pathway, resulting in an amplified production of ΔFosB mRNA within medium spiny neurons (MSNs) (Krapacher et al. 2022). Notably, this enhancement was mediated through the activation of PCBP1. When PCBP1 and SMAD3 were simultaneously activated by D1 and ALK4 signaling, they translocated into the cell nucleus, where they bound to specific sequences within exon 4 and intron 4 of the FosB mRNA, as detected by the RNA-proximity ligation assay. The disruption of either ALK4 or PCBP1 function in MSNs attenuated ΔFosB mRNA induction and the nuclear translocation of ΔFosB protein (Krapacher et al. 2022). These findings highlight the important role of PCBP1 in the alternative splicing of ΔFosB mRNA induced by dopamine D1 receptor agonism.

Taking into consideration the large repertoire of RBPs and their functional diversity during the splicing process, studies elucidating how drugs of abuse can affect the RBP-RNA regulatory network and how this impacts RNA splicing are essential. In this context, RNA immunoprecipitation (RIP) followed by high-throughput sequencing can reveal targets to which a particular type of RBP binds. The key question is whether exposure to substances such as alcohol, cocaine, or opioids alters these interactions. By generating a comprehensive pre-RNA binding and functional map of RBP changes in the presence and absence of these substances, we can identify the RBPs and their targets affected by drug exposure. Finally, functional studies that identify genes whose expression or splicing responds to perturbations in RBPs will generate hypothesis on the role of specific splice variants and splicing factors in behavior.

## Epigenetic modifications that influence RNA splicing

It has now become recognized that since splicing is a co-transcriptional process, chromatin can significantly impact the final splicing outcome (Agirre et al. 2021). The kinetic model suggests that chromatin states can slow down RNA polymerase II, thus increasing the time during which splicing regulators can bind to nascent RNA (Agirre et al. 2021). The recruitment model proposes that chromatin modifications, such as histone modifications and DNA methylation, can modulate the binding of splicing factors to pre-mRNA by recruiting chromatin-binding proteins that serve as adaptors between the chromatin and the splicing machinery (Luco et al. 2011). Drugs of abuse, such as cocaine and alcohol alter histone modifications and DNA methylation in key brain regions by altering the expression of histone- and DNA-modifying enzymes and metabolites required for histone acetylation and DNA methylation (Walker et al. 2015; Pandey et al. 2008; Feng et al. 2014; Lev Maor et al. 2015; Mews et al. 2019; Gatta et al. 2017). Therefore, another way in which drugs of abuse can potentially impact splicing is through the changes in epigenetic modifications at specific genes (Xu et al. 2021; Kyzar et al. 2019).

Epigenetic modifications can influence alternative splicing through various mechanisms. For instance, DNA methylation can impact splicing through promoter and intragenic methylation, influencing the recruitment of splicing regulators (Maunakea et al. 2013; Yearim et al. 2015). Histone modifications, such as acetylation, methylation, and ubiquitination, affect splicing by influencing splice site selection (Rahhal and Seto 2019; Hu et al. 2017). Chromatin remodeling complexes can directly alter splice site accessibility (Casteels et al. 2022). Non-coding RNAs, like microRNAs and lncRNAs, can act as splicing regulators or compete with splicing factors (Statello et al. 2021; Romero-Barrios et al. 2018; Stanek 2021). RNA modifications, such as N6-Methyladenosine (m6A), can influence splicing by recruiting or repelling splicing factors (Yang et al. 2018; Wang et al. 2022).

Feng et al. (2014) had previously examined combinations of histone modifications associated with transcript variant expression and found specific “chromatin signatures” correlated with cocaine-induced alternative splicing in the nucleus accumbens (NAc), a crucial brain region associated with cocaine-reward behavior. A subsequent study conducted in mice reported that cocaine injections induced changes in the enrichment of specific histone modifications in relation to various types of alternatively spliced exons within the nucleus accumbens (NAc) (Hu et al. 2017). Specifically, cocaine led to genome-wide differences in the enrichment of histone H3 lysine 36 trimethylation (H3K36me3), histone H3 lysine 27 trimethylation (H3K27me3), histone H3 lysine 9 dimethylation (H3K9me2), and histone H3 lysine 4 monomethylation (H3K4me1). Notably, H3K36me3 enrichment showed the strongest association with alternative splicing and served as a robust predictor for exon exclusion (Hu et al. 2017).

In another study, it was observed that both cocaine self-administration and the overexpression of SET2, a histone methyltransferase catalyzing the addition of methyl groups to H3K36, resulted in an overlap of alternative exons enriched for H3K36me3 and containing the binding motif for Srsf11, a serine and arginine-rich splicing factor 11 (Xu et al. 2021). Interestingly, the authors found that *Srsf11* mRNA itself underwent differential splicing and was enriched in H3K36me3 in mice treated with cocaine or SET2 overexpression. Targeted enrichment of H3K36me3 at *Srsf11* was achieved using a CRISPR-Cas9 method, by expressing nuclease-deficient Cas9 fused to the histone methyltransferase SET2 (dCas9-SET2) and *Srsf11* single guide (sg)RNA in the NAc. This replicated the cocaine-induced alternative splicing patterns and enhanced cocaine-reward behavior. These findings highlight the intricate relationship between drug-induced epigenetic modifications, their impact on splicing processes, and the subsequent effects on drug-related behavior.

While it has been established that alcohol exposure triggers changes in histone modifications and DNA methylation, the specific relationship of these epigenetic alterations to alcohol-induced alternative splicing remains to be determined (Pandey et al. 2008; Palmisano and Pandey 2017; Berkel and Pandey 2017). Similarly, it is known that chronic alcohol consumption alters the RNA methylome (m6A modification) (Liu and Zhang 2022), however, the contribution of this modification to changes in splicing and drug-related behavior remains to be investigated. Epigenetic marks can crosstalk, modulate transcription factor binding, alter 3D chromatin structure, exhibit cell-type-specific patterns, and collectively shaping context-dependent alternative splicing. Thus, this field of research is critical for understanding the complexity of gene regulation and its implications for behaviors related to addiction.

## Conclusions and future perspectives

Recent studies have highlighted that changes induced by drugs of abuse occur not only at the transcriptional level but also during RNA splicing. Current literature suggests that the impact of drugs of abuse on splicing can vary depending on factors such as the specific drug used, the duration and pattern of use, and individual genetics. Despite the progress made, numerous avenues for future exploration remain open. While genome-wide studies provide an overview of the components of the spliceosome altered due to drug exposure, there is a need for a deeper understanding of the precise molecular mechanisms through which drugs of abuse influence the splicing machinery and splicing factor expression. Furthermore, it is necessary to elucidate the functional consequences of drug-induced splicing changes for synaptic function and behavior. This can be achieved by knocking down targets via RNA interference (RNAi) or CRISPR/Cas9 methods followed by behavioral tests. Questions arise regarding whether alternative splicing contributes to drug-seeking behavior or anxiety and depression during drug withdrawal. Additionally, there is a need to examine the differences between acute and chronic effects of drug-induced splicing alterations. Understanding the consequences of drug exposure during developmental periods, such as adolescence, on alternative splicing in adulthood is essential. Moreover, exploring whether exposure to drugs of abuse leads to differential RNA splicing between males and females and whether this sex difference influences the risk of developing a SUD remains to be investigated.

Methods to study RNA splicing have undergone a transformative evolution. In the past, classical techniques primarily relied on PCR/RT-PCR and gel electrophoresis to analyze splicing patterns, often limiting the simultaneous examination of multiple splicing events. However, the emergence of high-throughput sequencing technologies, such as RNA-Seq, allows for a comprehensive and genome-wide analysis of alternative splicing events. Moreover, the recent integration of long-read sequencing technologies generates full-length transcripts, overcoming the challenges posed by short-read technologies in accurately identifying transcript isoforms (Leung et al. 2021; Pepke et al. 2009). Chromatin immunoprecipitation followed by sequencing (Chip-Seq), allows the investigation of the physical interactions between splicing regulators and chromatin regions associated with specific splicing events (Busch et al. 2020). Finally, RNA binding assays, such as RNA immunoprecipitation (RIP) or cross-linking immunoprecipitation (CLIP), followed by sequencing, allow researchers to identify the RNA molecules directly bound by splicing regulators at the whole transcriptome level. We predict that our knowledge of alternative splicing changes induced by drug exposure, and the behavioral consequences of these changes, will rapidly increase in the next several years.

## Abbreviations

AUD: Alcohol use disorder
RBP: RNA binding protein
SUD: Substance use disorder
snRNA: Small nuclear RNA
